# Evolution of postural control assessment: From dynamic posturography to virtual reality

**DOI:** 10.3389/fneur.2022.1054346

**Published:** 2023-01-11

**Authors:** Emily A. Keshner, Arthur I. Mallinson, Neil S. Longridge, Solara Sinno, Hannes Petersen, Philippe Perrin

**Affiliations:** ^1^Department of Health and Rehabilitation Sciences, Temple University, Philadelphia, PA, United States; ^2^Division of Otolaryngology, Department of Surgery, Faculty of Medicine, University of British Columbia, Vancouver, BC, Canada; ^3^Research Unit EA 3450 DevAH–Development, Adaptation and Handicap, Faculty of Medicine, University of Lorraine, Nancy, France; ^4^Laboratory for the Analysis of Posture, Equilibrium and Motor Function (LAPEM), University Hospital of Nancy, Nancy, France; ^5^Department of Otorhinolaryngology, University of Iceland, Reykjavík, Iceland; ^6^Department of Anatomy, Faculty of Medicine, University of Iceland, Reykjavik, Iceland

**Keywords:** balance, dizziness, vestibular rehabilitation, otoliths, diagnosis, intervention

## Abstract

During the early years of spaceflight it was documented that astronauts were impaired and incapacitated upon return to earth. Computerized Dynamic Posturography (CDP) was devised to investigate and quantify this deficit, and eventually progressed into a clinical assessment tool. The current sprouting of virtual reality (VR) technologies has allowed for the development of an alternative approach that could be more informative. Many low-cost VR systems (including desktop gaming programs designed for rehabilitation) are now available. Continued improvements in this technology indicate a high probability that VR will become an integral component of posturography by replacing present mechanical CDP techniques. We researched the relevant literature to evaluate the strengths and weaknesses of CDP using the Equitest (Neurocom International; Clackamas USA), and the added benefits of incorporating VR to help clinicians assess the complex task of balance maintenance. VR is capable of manipulating task and environmental demands in order to assess functional postural behavior. VR is also a useful tool for clinical testing of postural disorders resulting from sensory mismatch. Although posturography is still a useful clinical tool, VR provides an inherent conflict between the visual and vestibular senses and can elevate the effectiveness of CDP for both assessment and intervention. We conclude that, when initially developed, CDP was innovative and ahead of its time. However, with the advent of VR, we have a chance to modernize CDP and enhance its value as a clinical instrument.

## Introduction

The European Society for the Clinical Evaluation of Balance Disorders (ESCEBD), based in Nancy, France, has been meeting yearly since 2005 to discuss themes related to balance and equilibrium that are not yet clearly defined or standardized. One of our latest discussions was with regard to the continued development of Computerized Dynamic Posturography^Ⓡ^ (CDP) as a research and clinical tool in the clinical setting. Unfortunately, this method of testing is non-portable and expensive. We acknowledged that virtual reality (VR) has begun to emerge as a replacement for CDP from both the diagnostic and therapeutic points of view. The following is a brief synopsis of our discussion relating to the use of CDP in the clinical setting and how it has helped VR evolve as a clinical tool.

## Control of posture and balance

Posture can be defined as the control of neural circuitry over mechanics of the skeletal-motor system to maintain orientation in space, in response to momentary demands of a task in a dynamic environment. The progression of knowledge in this field of research has closely followed our understanding of central nervous system (CNS) processing and the dynamic interplay between the organism, the task, and the environment. The origin of research on the control of posture stems from the descriptions and categorizing of the reflexes that emerged when animals would orient themselves in space. Early research into the reflex control of posture from more than a century ago was based on studies of decerebrate and decorticate animals and focused primarily on the reflexes that positioned the body segments in space with respect to gravity (1).

A major theoretical shift occurred in the 1970's with the development of CDP, which enabled postural behaviors to be quantified during dynamic disturbances at the base of support. Attempts to model the mechanisms of posture control were done by simplifying the biomechanics of the behavior. Posture was primarily modeled as an inverted pendulum with the principal motion occurring around the ankle joint (2). Although the complexity of the human multisegmental and multimodal system makes it understandable that accurate modeling requires a reduction of variables, we need to acknowledge that a simplification strategy such as this may not be as robust or generalizable to natural motion as we might hope. Indeed, more complex models allowing for a greater number of variables have been developed that suggests that the system is more adaptive and variable than early controlled studies might suggest (3).

The balance system involves complex, adaptive interactions among multiple sensory and motor components. When pathology strikes one of these components, the balance system attempts to adapt by changing how other components contribute to the motor outcome. This adaptive process (i.e., “compensation”) can be difficult to untangle when several components have gradually modified through aging. Compensation is a multifactorial process that does not always reach a functional level. By the time a patient sees a medical professional for a balance problem, attempting to define or locate the site of pathology can be challenging. Even when the site of injury is identified, symptoms and impairments may vary widely across patients (4).

Balance and mobility deficits arise not only from the motor or sensory impairment but also from the inability to select and properly weight pertinent sensory information (5). If we accept that the human performer does not act as an inverted pendulum but rather as a multisegmental processor of the full array of incoming signals (6), then the additive approach to sensory signals that underlie a standard CDP system cannot produce functional assessments of balance. If we acknowledge that vestibular signs and symptoms can emerge from poorly weighted or conflicting multimodal signals (7, 8), then we must appreciate that VR presents more promise for a functional approach to treatment than a standard CDP system.

## Evolution of Computerized Dynamic Posturography

During the early years of spaceflight, NASA researchers reported that astronauts were unstable and nauseated upon their return to earth. It had originally been predicted that “… the symptoms [of motion sickness] are the same whether they result from the movement of ships, aircraft or cars” (9) and would also occur in space (10). Otoliths were thought to play a major role in space motion sickness (11) and the balance and autonomic/visceral control centers, traditionally viewed as separated, began to be viewed as one functional entity (12). Disrupted processing of otolith inputs upon return from orbital flight was thought to be the source of postural instability of astronauts. Impairments in returning astronauts were discussed and summarized as being of vestibular origin by Black et al. (13) and it has been proposed [e.g., (14)] that astronauts were impaired in the same manner as “vestibular patients.”

In order to investigate and quantify the vestibular deficit in returning astronauts, CDP was devised (15). This technology was commercialized as Equitest^Ⓡ^ (Neurocom International; Clackamas USA) in the mid-1980's and eventually, CDP was introduced as a clinical tool (16). CDP was the first diagnostic tool for the balance system that had been developed subsequent to the generally accepted vestibular tests (e.g., calorics which were described by Barany about 70 years previously). At the time CDP was developed, it was advanced technology in the clinical setting. Although expensive, systems were acquired by research laboratories in the field of balance and dizziness for both diagnostic and therapeutic purposes. Comparison of CDP investigations from one location to another made direct research between institutions possible; however, the prohibitive cost of CDP also led to a reliance on “home-made” force plate systems (e.g., “foam and dome”) (17). The drawback of these systems was that they were not reliable (foam degrades over time) and not standardized for their specific properties (e.g., compliance) across clinical institutions.

CDP allows us to assess postural performance when challenged with the task of maintaining balance under situations which are orientationally disruptive. This allows for measurement of how *well* the patient is compensating, but also *how* they are doing so. The whole process of compensation can itself be a challenge, as performers have been shown to select information based on individual sensory preferences (6). CDP is very helpful at documenting subtle pathology, especially in the atypical patient (18).

## Benefits of CDP to vestibular rehabilitation

Although a sensitive and standardized assessment technique, CDP is neither site-specific nor side specific. Unfortunately, the cost for CDP remains high and is prohibitive in terms of general availability. As time has passed and newer tests have developed, the role of CDP in the diagnostic toolbox has become less secure.

Despite these limitations, CDP should be recognized for its role in assessing patients with vestibular dysfunction. Only 10 to 20% of patients with dizziness suggestive of vestibular dysfunction have abnormalities on caloric testing as the low frequency caloric stimulus does not sufficiently challenge the vestibular system (19). Patients may exhibit symptoms of unexplained motion sickness that is not related to semicircular canal pathology and caloric testing is only capable of assessing the lateral semicircular canals. With CDP, abnormalities have been measured in about 50% of these patients (20). Thus, CDP results are a more robust indicator of a vestibular deficit.

Complaints of dizziness which are sometimes regarded as “nontraditional” in nature are strongly suggestive of otolithic pathology, and there is evidence that CDP effectively identifies impairment of otolithic structures (21, 22). Astronauts with similar impairments post flight also exhibit abnormal posturography and autonomic symptoms. These abnormalities are physiologic and suggest pathology of the balance system, quite possibly related to a disruptive effect on otolithic inputs (13, 14). The specificity for detection of “otolith disorders” is unclear (22), however. It has been stressed that otolithic disorders affect posture, but postural tests themselves do not specifically measure otolithic function. This has become important as otolithic disorders can occur in an isolated fashion without other pathology (23). Individuals who have suffered head and neck trauma can also exhibit otolithic symptoms and it has been shown that the results of assessments in two different populations (i.e., vestibular dysfunction as a result of trauma and without trauma) are the same (4, 21, 24). A wide range of pharmacotherapeutics is available to manage symptoms of dizziness (18), but there are none that relieve the underlying cause. Cognitive behavioral therapy combined with traditional vestibular physical therapy has offered some relief to those experiencing anxiety-related dizziness, but no lasting effect has been demonstrated (25).

The vestibular system may also generate symptoms of dizziness in some earth-bound individuals similar to those of returning astronauts even in the absence of balance difficulty. These individuals are often categorized as being visually sensitive or having visual dependence (2, 19, 21). This symptom complex has been recently grouped under the term persistent postural-perceptual dizziness or PPPD (25). A significant obstacle to the development of a standard treatment protocol for individuals with PPPD is that they do not always demonstrate vestibular dysfunction with standard clinical testing.

Individuals who have been given a diagnosis of PPPD for their symptom complex often state after a CDP assessment that the assessment protocol has reproduced their symptom set. This report from a patient is very helpful diagnostically, as it suggests the symptoms are of vestibular origin (24, 26). In addition to helping localize the pathology, it is also often a great relief to the individual sufferer, who may be concerned that their complaints will be dismissed. It is not uncommon for patients to hear that “since all the assessments are normal there probably isn't anything wrong with you.” This leads to wrongly categorizing their complaints as psychiatric in origin (18). No other assessment techniques have been shown to simulate and characterize this symptom set, thus CDP remains a valuable clinical tool.

## Benefits of VR to vestibular rehabilitation

Vision is a powerful input to the balance system, and its role as a sensory input in balance maintenance is well assessed by CDP. Disturbances of posture and spatial orientation via visual motion and complexity are, however, due to perceptual as well as vestibular influences (27–29) which can not be fully controlled with CDP. Several studies have demonstrated that posture could be functionally changed after repeated exposures to intense optokinetic stimulation (30, 31) or visually busy virtual environments (32, 33). The limiting factor to this approach has been the tolerance of an individual to the treatment which often exacerbates negative symptoms of nausea and dizziness (27, 33).

In order to assess the role played by the vision in everyday environmental situations, it is useful to expose the patient to a wide variety of visual conditions relevant to daily life activities. Although this is not an option provided by a standard CDP system, the tremendous advances in computer graphics technology and the development of VR now afford us the ability to re-create specific visual environments and also change the properties of a visual scene to reduce or enhance object “clutter,” alter positions of objects, and change colors and contrast. These are all properties that influence motion perception within the visual environment. A visual scene can also be programmed to move in-phase or out-of-phase with the natural movements of the patient. All these capabilities can be of value when the patient is being assessed to objectively define subjective reports of dizziness. Once the instrumental disturbance is identified, the individual can be trained to cope with existing deficits. Still to be explored is whether it is more effective to gradually expose the system to disturbing stimuli in order to provoke neural adaptation (34), or to disrupt the system with intense stimulation (19, 20, 22, 35, 36) to provoke desensitization and habituation. Both approaches can be addressed with VR.

Neither visual or vestibular signals are exclusively responsible for perceptual disturbances of balance and spatial orientation. Indeed, the ability to separate self-motion from motion of the world is dependent on the confluence of visual-vestibular information. Spatial disorientation is evident during an illusion of self-motion and even during self-initiated motion when a full field of view visual motion does not match actual or imagined physical motion (37). Positron emission tomography (PET) and magnetic resonance imaging (MRI) studies indicate that when both retinal and vestibular inputs are processed, there are changes in the medial parieto-occipital visual area and parieto-insular vestibular cortex (38, 39) as well as the cerebellar nodulus (28, 34) suggesting the nervous system deactivates the structures that process object-motion when there is a perception of physical motion.

VR is an excellent tool for the clinical assessment and intervention of postural disorders resulting from sensory mismatch. As stated above, the value of VR in assessment lies in its flexible presentation of complex and demanding visual environments. A recent study (40) has demonstrated that the presence or absence of recognizable objects and verticality cues in the visual world influences the attainment of postural control and spatial orientation in individuals diagnosed with visual-vestibular mismatch disorders.

VR can portray unexpected circumstances, such as a tilting room, producing a mismatch between the world and physical motion. This produces a sensory conflict resulting in the inability to distinguish between visual motion and self-motion (41, 42). A virtual optic flow field not matched to the performer's head motion produces disparity between visual and vestibular inputs that results in a perception of self-motion called vection (32). Extensive work in the literature, ranging from behavioral studies (31, 33, 43) to neuroimaging studies (27, 34, 38) has demonstrated that vestibular and visual inputs converge at the brainstem and cortical levels during vection to contribute to the illusion of self-motion. Current approaches to the treatment of perceptual-vestibular complaints rely on desensitization to such erroneous or conflicting visual cues that occur in VR (30, 35, 36, 44).

But it also needs to be acknowledged that VR alone is not sufficient for assessment of postural dysfunction. CDP is a reliable measure of postural instability but VR requires additional technologies (e.g., motion analysis and EMG systems) to measure meaningful change in motor control mechanisms (45, 46). The weakness of VR as an independent measure of postural stability was revealed in an intervention protocol performed by Drs. Keshner, Longridge and Mallinson at Vancouver General Hospital (unpublished data). Using standardized clinical tools, they attempted to chart changes in postural control and disorientation in four individuals (37–60 yrs) who had complained of chronic symptoms of dizziness and nausea. Each participant stood on dense foam while wearing an Oculus Rift (https://www.oculus.com) and viewing a virtual environment with no cues to vertical once a week for four weeks. The visual field was rotated sinusoidally in pitch and roll and anterior-posterior translated at 0.25 Hz for 60 s, 3 times in each direction. The field of view (FOV) was initially presented as central visual field stimulation of +/- 10 deg horizontal and gradually increased over the four weeks. Outcome measures were taken following a pre-intervention and post-intervention trial with a more complexly textured environment.

**Figure 1:**
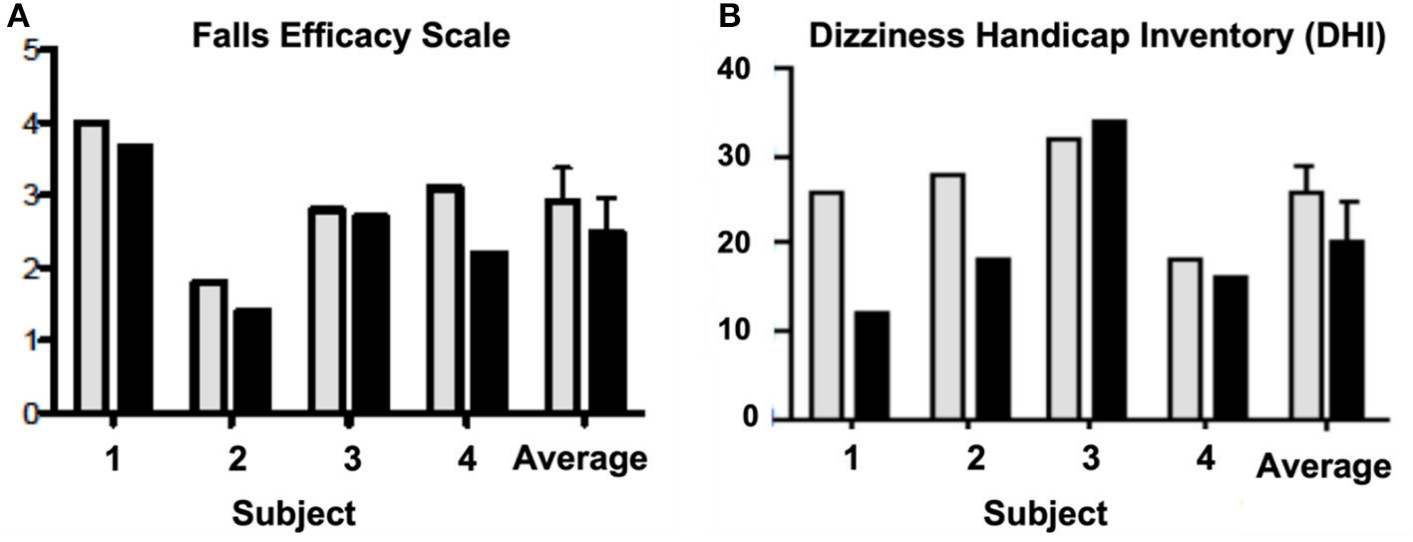
Pre-test (gray) and post-test (black) scores on the **(A)** Falls Efficacy Scale (47) and the **(B)** Dizziness Handicap Inventory (DHI) (48) for each subject. The last two columns on each graph are the average scores + SD across all four subjects for the pre- and post-test.

Outcome measures suggested that exposure to the virtual environment had a positive impact on postural control, but the investigators were disheartened by the variability of results in patients with apparently similar symptoms. Participants fell on 75% of the pre-test trials whereas only one fall occurred during post-testing, but this was not reflected on the Falls Efficacy Scale results (Figure 1). Three of four subjects reported increased balance confidence and reductions in subjective dizziness. However, these results relied on subjective reporting and could have been due to practice or individualized attention. There was no way to verify that gradually increasing exposure to conflicting visual and self-motion feedback produced adaptation or habituation. To do that, a more objective measurement technology, such as CDP, would need to be incorporated into this protocol (49).

## Reinventing CDP in the 21^st^ century

Initial attempts to measure and quantify balance maintenance through CDP revealed automatic postural reactions that exhibited shorter latencies than voluntary behaviors but were also adaptive and modifiable (16) depending on the parameters of the postural disturbance and the particular abilities of the individual. Thus, posture maintenance was considered a process that was learned and became automatic through repeated use, but also depended primarily upon the nature of the task at hand. It was not thought to be influenced by any pre-planning or attentional resources of the performer. Scientific and clinical interest focused primarily on the sensory pathways controlling and generating postural reactions (2, 3) and how the loss of these pathways would alter postural behavior.

The greatest value of CDP as a diagnostic tool is that it quantifies how a person is using their balance system to cope with different sensory inputs even though the role played by the vestibular system in balance maintenance, and what happens when something goes wrong with the system, is still not well documented. The development of CDP supplied the tools for the clinician to document and quantify abnormalities in patients who up until this point were diagnosed based on their subjectively reported symptomatic history alone. CDP could be used to measure static sway and also increase the overall diagnostic sensitivity of vestibular function testing. CDP can also be used to design a balance training program to help address a motor behavior deficit and monitor the progression of clinical recovery. The current absence of reliable inter-institutional comparisons might be assuaged by incorporating an inexpensive, reliability tested hard CDP like standard force plate which could potentially reduce the years of contradictory studies necessary to find the most effective therapeutic protocols.

When initially developed, CDP was innovative and ahead of its time. However, as new technologies and better computerization have developed, the advent of VR gives an opportunity to modernize CDP so that it continues to be a valuable clinical tool. The principle behind VR is its ability to produce a disturbance in spatial orientation through perception of the visual environment, rather than mechanical instability at the base of support. Thus, incorporating VR as a tool for assessing and treating balance disorders may shift the focus of clinical assessment from the maintenance of biomechanical stability to the assessment of whole body balance and orientation behaviors (46). Currently, a number of low-cost VR systems designed for and targeting rehabilitation have become available. These include a variety of rehabilitation-oriented desktop gaming programs that implement VR properties (e.g., feedback, documentation, motivation). The increasing accessibility of embedded ambient technologies (e.g., inexpensive cameras, proximity sensors, wearable computing) that support the monitoring of motor and cognitive functioning under real-world conditions has extended VR-based interventions beyond the clinical setting (50). Evidence is also accumulating that learning in the virtual environment will transfer to the physical world (29, 51, 52). Through employing the principles of motor learning (53, 54) and adding tools such as robots, treadmills, and dynamic platforms into the virtual environment, we can manipulate task and environmental demands in order to assess meaningful postural behavior. Continued improvements in this technology indicate a high probability that VR will become an integral component of (and possibly supplant) CDP (50) to provide valued diagnostic and therapeutic information to the clinician.

## Discussion

The broad range of postural control and motor abilities among both healthy and impaired individuals necessitates that test conditions be adapted to individual abilities. This would require the development of various test conditions and comparison of the performance within each individual, which can be achieved with VR. Similar to the combination of optokinetic stimulation and VR, combining CDP with VR should assist with rehabilitation by inducing symptoms and encouraging “habituation.” CDP and VR are also helpful in documenting and reproducing patient symptoms, which will aid the clinician to understand the presenting complaints and perform, through the use of these tools, optimal rehabilitation. We maintain that combining CDP with VR provides the strongest available approach to assessment and intervention for postural and orientation disorders.

## Data availability statement

The original contributions presented in the study are included in the article/supplementary material, further inquiries can be directed to the corresponding author.

## Ethics statement

The studies involving human participants were reviewed and approved by University of Vancouver IRB. The patients/participants provided their written informed consent to participate in this study.

## Author contributions

EK, AM, and PP were responsible for the content of the paper. NL, SS, and HP were responsible for editing and approving the content. All authors participated as speakers at the Clinical Evaluation of Balance Disorders (ESCEBD) meeting in Nancy, France and contributed to the conceptualization of this paper.
